# Poly[dimethyl­ammonium [aquadi-μ_2_-oxalato-yttriate(III)] trihydrate]

**DOI:** 10.1107/S1600536811019209

**Published:** 2011-06-04

**Authors:** Yao-Kang Lv, Li-Hua Gan, Liang Xu, Hao-Wen Zheng, Cao Liu

**Affiliations:** a Department of Chemistry, Tongji University, Shanghai 200092, People’s Republic of China

## Abstract

The title complex, {(C_2_H_8_N)[Y(C_2_O_4_)_2_(H_2_O)]·3H_2_O}_*n*_, was obtained accidentally under hydro­thermal conditions. The Y^III^ atom is chelated by four oxalate ligands and one water mol­ecule resulting in a distorted tricapped trigonal–prismatic geometry. Each oxalate ligand bridges two Y^III^ atoms, thus generating a three-dimensional network with cavities in which the ammonium cations and lattice water mol­ecules reside. Various O—H⋯O and N—H⋯O hydrogen-bonding inter­actions stabilize the crystal structure. The title complex is isotypic with the Eu and Dy analogues.

## Related literature

For general background to the rational design and synthesis of metal-organic polymers, see: Lv *et al.* (2010 ▶, 2011 ▶). For related structures, see: Platel *et al.* (2009 ▶); Gao & Cui (2008 ▶); Deguenon *et al.* (1990 ▶). The structure of the isotypic Eu^III^ compound was reported by Yang *et al.* (2005 ▶), and that of the Dy^III^ compound by Ye & Lin (2010 ▶). For decomposition products obtained under hydro­thermal conditions, see: Song *et al.* (2004 ▶).
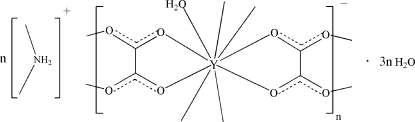


## Experimental

### 

#### Crystal data

(C_2_H_8_N)[Y(C_2_O_4_)_2_(H_2_O)]·3H_2_O
*M* *_r_* = 383.11
Monoclinic, 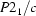

*a* = 9.6008 (1) Å
*b* = 11.5422 (2) Å
*c* = 14.2886 (2) Å
β = 122.460 (1)°
*V* = 1336.00 (3) Å^3^
*Z* = 4
Mo *K*α radiation
μ = 4.43 mm^−1^
*T* = 293 K
0.31 × 0.20 × 0.19 mm

#### Data collection

Bruker APEXII area-detector diffractometer
Absorption correction: multi-scan (*SADABS*; Sheldrick, 1996 ▶) *T*_min_ = 0.36, *T*_max_ = 0.43
11935 measured reflections
3040 independent reflections
2384 reflections with *I* > 2σ(*I*)
*R*_int_ = 0.044

#### Refinement

*R*[*F* ^2^ > 2σ(*F* ^2^)] = 0.033
*wR*(*F* ^2^) = 0.082
*S* = 1.00
3040 reflections
207 parameters
13 restraints
H atoms treated by a mixture of independent and constrained refinement
Δρ_max_ = 0.70 e Å^−3^
Δρ_min_ = −0.58 e Å^−3^

### 

Data collection: *APEX2* (Bruker, 2006 ▶); cell refinement: *SAINT* (Bruker, 2006 ▶); data reduction: *SAINT*; program(s) used to solve structure: *SHELXS97* (Sheldrick, 2008 ▶); program(s) used to refine structure: *SHELXL97* (Sheldrick, 2008 ▶); molecular graphics: *DIAMOND* (Brandenburg & Putz, 2004 ▶); software used to prepare material for publication: *SHELXTL* (Sheldrick, 2008 ▶).

## Supplementary Material

Crystal structure: contains datablock(s) global, I. DOI: 10.1107/S1600536811019209/wm2489sup1.cif

Structure factors: contains datablock(s) I. DOI: 10.1107/S1600536811019209/wm2489Isup2.hkl

Additional supplementary materials:  crystallographic information; 3D view; checkCIF report

## Figures and Tables

**Table 1:** Hydrogen-bond geometry (Å, °)

*D*—H⋯*A*	*D*—H	H⋯*A*	*D*⋯*A*	*D*—H⋯*A*
O1*W*—H1*WA*⋯O2*W*^i^	0.83 (2)	1.93 (2)	2.742 (4)	167 (4)
O1*W*—H1*WB*⋯O2*W*^ii^	0.72 (2)	2.20 (2)	2.861 (4)	152 (4)
O2*W*—H2*WA*⋯O6^iii^	0.81 (2)	2.38 (2)	3.143 (4)	158 (5)
O2*W*—H2*WA*⋯O7^iv^	0.81 (2)	2.45 (5)	2.944 (4)	121 (5)
O2*W*—H2*WB*⋯O3*W*^v^	0.79 (2)	2.38 (3)	2.963 (7)	131 (4)
O2*W*—H2*WB*⋯O4*W*	0.79 (2)	2.44 (3)	3.194 (6)	159 (5)
O3*W*—H3*WA*⋯O2	0.88 (2)	2.36 (7)	2.830 (5)	114 (6)
O3*W*—H3*WB*⋯O4*W*^iv^	0.86 (2)	1.90 (3)	2.735 (6)	161 (6)
O4*W*—H4*WA*⋯O1^vi^	0.82 (2)	2.13 (2)	2.943 (4)	172 (5)
O4*W*—H4*WB*⋯O3^vii^	0.83 (2)	2.08 (3)	2.857 (4)	155 (6)
N1—H1*A*⋯O8^vi^	0.90	2.00	2.869 (4)	163
N1—H1*A*⋯O1*W*^vi^	0.90	2.54	3.107 (4)	122
N1—H1*B*⋯O3*W*	0.90	1.90	2.784 (6)	166

Symmetry codes: (i) 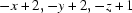; (ii) 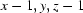; (iii) 
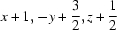
; (iv) 
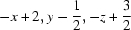
; (v) 
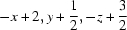
; (vi) 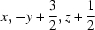; (vii) 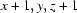.

**Fig. 1.**
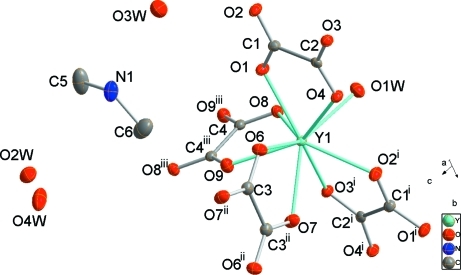
An expanded vioew of the asymmetric unit of (I), showing the coordination of the YIII atom, and the presence of the lattice water molecules and the ammonium cation. All hydrogen atoms were omitted for clarity. [Symmetry codes: (i) -x + 1, y + 1/2, -z + 1/2; (ii) -x + 1, -y + 2, -z + 1; (iii) -x + 2, -y + 2, -z + 1.]

**Fig. 2.**
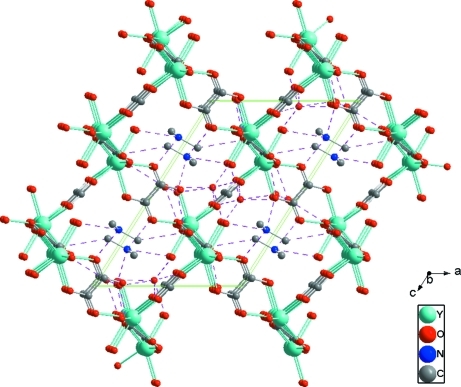
View of the three-dimensional framework of (I). All hydrogen atoms are omitted for clarity. Hydrogen bonding between donator and acceptor atoms is indicated by dashed lines.

## supplementary crystallographic information

### Comment

Rational design and synthesis of metal-organic polymers have attracted much attention in the field of supramolecular chemistry and crystal engineering (Lv *et al.*, 2010; 2011). Oxalate, which usually represent one of the products of the degradation of some organic compounds, is one of the simplest multidentate organic ligands potentially able to bridge metal ions in a bidentate chelating manner (Deguenon *et al.*, 1990). Herein, we report the synthesis and structure of a novel yttrium(III) complex, (C_2_H_8_N)[Y(C_2_O_4_)_2_(H_2_O)]^.^3H_2_O, (I).

Complex (I) is isotypic with its Eu(III) (Yang *et al.*, 2005) and Dy(III) (Ye & Lin, 2010) analogues. As shown in Fig. 1, the Y^III^ atom is chelated by four oxalate ligands and one water molecule resulting in a distorted tricapped trigonal-prismatic coordination environment. The Y—O bond lengths fall in the range of 2.374 (2)-2.459 (2) Å, which is in agreement with comparable values reported elsewhere (Platel *et al.*, 2009; Gao & Cui, 2008). Each oxalate ligand bridges two Y^III^ atoms, thus generating a three-dimensional network with cavities where the ammonium cations and lattice water molecules reside (Fig. 2). Furthermore, there are various hydrogen-bonding interactions (N—H···O and O—H···O), involving the lattice water molecules and the cations, which give rise to a tightly held network structure.

### Experimental

A mixture of D-saccharic acid potassium salt (0.248 g, 1.0 mmol), Y(NO_3_)_3_^.^6H_2_O (0.191 g, 0.5 mmol) and N,N-dimethylformamide (20 ml) was stirred and heated at 373 K for 1 hour. The resulted colorless solution was kept at 293 K. Colorless block-shaped crystals of the title compound suitable for X-ray crystallographic study were obtained via slow evaporation within 2 weeks.

It is most likely that the oxalate ligands in this complex originates from the decomposition of the potassium salt of D-saccharic acid, and the protonated dimethylamine cations compensating the negative charge of the anionic network are believed to result from decomposition of the N,N-dimethylformamide solvent (Song *et al.*, 2004; Ye & Lin, 2010).

### Refinement

The hydrogen atoms attached to carbon and nitrogen atoms were positioned geometrically, while those attached to oxygen atom were located from difference Fourier maps. H atoms attached to C atoms were refined using a riding model with C—H = 0.96 Å and U_iso_(H) = 1.2U_eq_(C); H atoms attached to N atoms were refined with N—H = 0.90 Å and U_iso_(H) = 1.2U_eq_(N); H atoms attached to O atoms were refined without distance restraints and with U_iso_(H) = 1.2U_eq_(O).

### Crystal data

**Table:**

(C_2_H_8_N)[Y(C_2_O_4_)_2_(H_2_O)]·3H_2_O	*F*(000) = 776
*M**_r_* = 383.11	*D*_x_ = 1.905 Mg m^−^^3^
Monoclinic, *P*2_1_/*c*	Mo *K*α radiation, λ = 0.71073 Å
Hall symbol: -P 2ybc	Cell parameters from 2287 reflections
*a* = 9.6008 (1) Å	θ = 2.0–28.0°
*b* = 11.5422 (2) Å	µ = 4.43 mm^−^^1^
*c* = 14.2886 (2) Å	*T* = 293 K
β = 122.460 (1)°	Block, colourless
*V* = 1336.00 (3) Å^3^	0.31 × 0.20 × 0.19 mm
*Z* = 4	

### Data collection

**Table:**

Bruker APEXII area-detector diffractometer	3040 independent reflections
Radiation source: fine-focus sealed tube	2384 reflections with *I* > 2σ(*I*)
graphite	*R*_int_ = 0.044
ω scans	θ_max_ = 27.5°, θ_min_ = 2.9°
Absorption correction: multi-scan (*SADABS*; Sheldrick, 1996)	*h* = −12→12
*T*_min_ = 0.36, *T*_max_ = 0.43	*k* = −14→14
11935 measured reflections	*l* = −18→18

### Refinement

**Table:**

Refinement on *F*^2^	Primary atom site location: structure-invariant direct methods
Least-squares matrix: full	Secondary atom site location: difference Fourier map
*R*[*F*^2^ > 2σ(*F*^2^)] = 0.033	Hydrogen site location: inferred from neighbouring sites
*wR*(*F*^2^) = 0.082	H atoms treated by a mixture of independent and constrained refinement
*S* = 1.00	*w* = 1/[σ^2^(*F*_o_^2^) + (0.0359*P*)^2^ + 1.2217*P*] where *P* = (*F*_o_^2^ + 2*F*_c_^2^)/3
3040 reflections	(Δ/σ)_max_ = 0.001
207 parameters	Δρ_max_ = 0.70 e Å^−^^3^
13 restraints	Δρ_min_ = −0.58 e Å^−^^3^

### Special details

**Table:**

Geometry. All e.s.d.'s (except the e.s.d. in the dihedral angle between two l.s. planes) are estimated using the full covariance matrix. The cell e.s.d.'s are taken into account individually in the estimation of e.s.d.'s in distances, angles and torsion angles; correlations between e.s.d.'s in cell parameters are only used when they are defined by crystal symmetry. An approximate (isotropic) treatment of cell e.s.d.'s is used for estimating e.s.d.'s involving l.s. planes.
Refinement. Refinement of *F*^2^ against ALL reflections. The weighted *R*-factor *wR* and goodness of fit *S* are based on *F*^2^, conventional *R*-factors *R* are based on *F*, with *F* set to zero for negative *F*^2^. The threshold expression of *F*^2^ > σ(*F*^2^) is used only for calculating *R*-factors(gt) *etc*. and is not relevant to the choice of reflections for refinement. *R*-factors based on *F*^2^ are statistically about twice as large as those based on *F*, and *R*- factors based on ALL data will be even larger.

### Fractional atomic coordinates and isotropic or equivalent isotropic displacement parameters (Å^2^)

**Table:**

	*x*	*y*	*z*	*U*_iso_*/*U*_eq_	
Y1	0.61770 (3)	0.98846 (2)	0.33257 (2)	0.01817 (10)	
O1	0.7049 (3)	0.78990 (17)	0.34286 (19)	0.0270 (5)	
O2	0.6167 (3)	0.60733 (18)	0.2950 (2)	0.0304 (6)	
O3	0.3054 (3)	0.68334 (17)	0.17013 (18)	0.0231 (5)	
O4	0.3959 (3)	0.86535 (17)	0.20743 (19)	0.0259 (5)	
O6	0.5198 (3)	0.88268 (18)	0.4319 (2)	0.0293 (5)	
O7	0.5387 (3)	1.11238 (19)	0.4367 (2)	0.0320 (6)	
O8	0.8892 (3)	0.99123 (17)	0.35640 (19)	0.0245 (5)	
O9	0.8389 (3)	0.98964 (18)	0.52152 (19)	0.0278 (5)	
O1W	0.6044 (3)	0.9808 (2)	0.1578 (2)	0.0348 (6)	
H1WA	0.617 (5)	1.039 (2)	0.129 (3)	0.042*	
H1WB	0.568 (4)	0.931 (2)	0.122 (3)	0.042*	
O2W	1.3774 (5)	0.8468 (3)	0.9672 (3)	0.0649 (10)	
H2WA	1.386 (6)	0.785 (3)	0.946 (4)	0.078*	
H2WB	1.293 (4)	0.850 (4)	0.965 (5)	0.078*	
O3W	0.8383 (7)	0.5082 (4)	0.5055 (4)	0.1038 (17)	
H3WA	0.857 (10)	0.539 (5)	0.457 (5)	0.125*	
H3WB	0.889 (8)	0.444 (3)	0.512 (6)	0.125*	
O4W	1.0523 (5)	0.7844 (3)	0.9661 (3)	0.0861 (13)	
H4WA	0.959 (3)	0.758 (5)	0.936 (4)	0.103*	
H4WB	1.103 (6)	0.758 (5)	1.030 (2)	0.103*	
N1	0.9245 (4)	0.6326 (3)	0.6962 (3)	0.0585 (11)	
H1A	0.8928	0.6007	0.7394	0.070*	
H1B	0.8808	0.5895	0.6341	0.070*	
C1	0.5960 (4)	0.7148 (2)	0.2919 (3)	0.0222 (7)	
C2	0.4163 (4)	0.7587 (2)	0.2166 (3)	0.0195 (6)	
C3	0.4947 (4)	0.9335 (3)	0.4984 (3)	0.0243 (7)	
C4	1.0151 (4)	1.0005 (2)	0.4524 (3)	0.0202 (6)	
C5	1.0993 (6)	0.6253 (6)	0.7531 (5)	0.0864 (19)	
H5A	1.1314	0.5459	0.7567	0.104*	
H5B	1.1476	0.6553	0.8269	0.104*	
H5C	1.1370	0.6699	0.7141	0.104*	
C6	0.8547 (8)	0.7488 (5)	0.6652 (5)	0.0897 (19)	
H6A	0.7365	0.7442	0.6254	0.108*	
H6B	0.8872	0.7841	0.6190	0.108*	
H6C	0.8944	0.7946	0.7309	0.108*	

### Atomic displacement parameters (Å^2^)

**Table:**

	*U*^11^	*U*^22^	*U*^33^	*U*^12^	*U*^13^	*U*^23^
Y1	0.01775 (15)	0.01434 (15)	0.01968 (16)	−0.00007 (11)	0.00824 (12)	−0.00024 (11)
O1	0.0193 (12)	0.0194 (11)	0.0322 (14)	−0.0013 (8)	0.0071 (11)	−0.0044 (9)
O2	0.0264 (12)	0.0173 (11)	0.0332 (14)	0.0029 (9)	0.0066 (11)	−0.0002 (9)
O3	0.0203 (11)	0.0193 (10)	0.0265 (13)	−0.0017 (8)	0.0105 (10)	−0.0016 (9)
O4	0.0232 (12)	0.0170 (10)	0.0285 (13)	0.0016 (8)	0.0079 (10)	0.0003 (9)
O6	0.0347 (13)	0.0252 (12)	0.0331 (15)	−0.0039 (9)	0.0216 (12)	−0.0047 (10)
O7	0.0424 (14)	0.0252 (12)	0.0402 (16)	0.0045 (10)	0.0301 (13)	0.0060 (10)
O8	0.0195 (10)	0.0307 (12)	0.0205 (11)	0.0009 (9)	0.0088 (9)	−0.0019 (9)
O9	0.0209 (11)	0.0368 (13)	0.0265 (12)	−0.0014 (9)	0.0132 (10)	−0.0004 (10)
O1W	0.0408 (14)	0.0346 (14)	0.0265 (14)	−0.0076 (12)	0.0165 (12)	−0.0043 (11)
O2W	0.080 (3)	0.0390 (17)	0.069 (2)	0.0025 (16)	0.036 (2)	0.0072 (16)
O3W	0.112 (4)	0.095 (3)	0.061 (3)	0.031 (3)	0.017 (3)	−0.013 (2)
O4W	0.070 (3)	0.061 (2)	0.069 (3)	−0.0094 (19)	−0.001 (2)	0.0154 (19)
N1	0.045 (2)	0.071 (3)	0.058 (3)	−0.0039 (18)	0.027 (2)	0.022 (2)
C1	0.0233 (16)	0.0187 (15)	0.0242 (17)	0.0003 (11)	0.0124 (14)	−0.0014 (12)
C2	0.0236 (17)	0.0206 (15)	0.0165 (16)	−0.0012 (11)	0.0121 (14)	−0.0026 (12)
C3	0.0184 (14)	0.0272 (17)	0.0257 (17)	−0.0012 (12)	0.0108 (13)	−0.0008 (13)
C4	0.0205 (14)	0.0145 (14)	0.0222 (16)	0.0004 (11)	0.0092 (13)	−0.0013 (12)
C5	0.046 (3)	0.144 (6)	0.066 (4)	−0.004 (3)	0.028 (3)	0.027 (4)
C6	0.110 (5)	0.073 (4)	0.103 (5)	0.024 (3)	0.068 (4)	0.029 (3)

### Geometric parameters (Å, °)

**Table:**

Y1—O3^i^	2.374 (2)	O2W—H2WA	0.805 (19)
Y1—O9	2.376 (2)	O2W—H2WB	0.794 (19)
Y1—O4	2.380 (2)	O3W—H3WA	0.88 (2)
Y1—O6	2.413 (2)	O3W—H3WB	0.86 (2)
Y1—O1	2.417 (2)	O4W—H4WA	0.82 (2)
Y1—O2^i^	2.422 (2)	O4W—H4WB	0.83 (2)
Y1—O1W	2.432 (3)	N1—C5	1.421 (6)
Y1—O8	2.441 (2)	N1—C6	1.458 (6)
Y1—O7	2.459 (2)	N1—H1A	0.9000
O1—C1	1.247 (3)	N1—H1B	0.9000
O2—C1	1.254 (3)	C1—C2	1.548 (4)
O2—Y1^ii^	2.422 (2)	C3—O7^iii^	1.250 (4)
O3—C2	1.254 (3)	C3—C3^iii^	1.536 (6)
O3—Y1^ii^	2.374 (2)	C4—O9^iv^	1.248 (4)
O4—C2	1.243 (3)	C4—C4^iv^	1.535 (6)
O6—C3	1.246 (4)	C5—H5A	0.9600
O7—C3^iii^	1.250 (4)	C5—H5B	0.9600
O8—C4	1.255 (4)	C5—H5C	0.9600
O9—C4^iv^	1.248 (4)	C6—H6A	0.9600
O1W—H1WA	0.828 (18)	C6—H6B	0.9600
O1W—H1WB	0.720 (17)	C6—H6C	0.9600
			
O3^i^—Y1—O9	85.24 (7)	C3—O6—Y1	120.4 (2)
O3^i^—Y1—O4	135.57 (7)	C3^iii^—O7—Y1	119.2 (2)
O9—Y1—O4	138.66 (8)	C4—O8—Y1	118.9 (2)
O3^i^—Y1—O6	135.37 (8)	C4^iv^—O9—Y1	121.0 (2)
O9—Y1—O6	74.17 (8)	Y1—O1W—H1WA	122 (3)
O4—Y1—O6	70.48 (8)	Y1—O1W—H1WB	119 (3)
O3^i^—Y1—O1	143.01 (7)	H1WA—O1W—H1WB	117 (3)
O9—Y1—O1	82.37 (7)	H2WA—O2W—H2WB	110 (3)
O4—Y1—O1	67.70 (7)	H3WA—O3W—H3WB	95 (7)
O6—Y1—O1	73.50 (8)	H4WA—O4W—H4WB	106 (3)
O3^i^—Y1—O2^i^	67.82 (7)	C5—N1—C6	115.9 (5)
O9—Y1—O2^i^	139.01 (8)	C5—N1—H1A	108.3
O4—Y1—O2^i^	71.18 (7)	C6—N1—H1A	108.3
O6—Y1—O2^i^	103.39 (8)	C5—N1—H1B	108.3
O1—Y1—O2^i^	137.25 (7)	C6—N1—H1B	108.3
O3^i^—Y1—O1W	82.13 (8)	H1A—N1—H1B	107.4
O9—Y1—O1W	133.57 (8)	O1—C1—O2	126.8 (3)
O4—Y1—O1W	70.99 (8)	O1—C1—C2	116.8 (2)
O6—Y1—O1W	139.75 (8)	O2—C1—C2	116.4 (3)
O1—Y1—O1W	81.59 (8)	O4—C2—O3	126.2 (3)
O2^i^—Y1—O1W	74.46 (9)	O4—C2—C1	116.8 (3)
O3^i^—Y1—O8	70.84 (7)	O3—C2—C1	117.0 (3)
O9—Y1—O8	66.75 (7)	O6—C3—O7^iii^	126.7 (3)
O4—Y1—O8	124.78 (8)	O6—C3—C3^iii^	117.1 (4)
O6—Y1—O8	130.44 (8)	O7^iii^—C3—C3^iii^	116.2 (4)
O1—Y1—O8	72.24 (7)	O9^iv^—C4—O8	127.1 (3)
O2^i^—Y1—O8	126.06 (8)	O9^iv^—C4—C4^iv^	116.9 (3)
O1W—Y1—O8	66.89 (8)	O8—C4—C4^iv^	116.0 (3)
O3^i^—Y1—O7	70.02 (7)	N1—C5—H5A	109.5
O9—Y1—O7	71.75 (8)	N1—C5—H5B	109.5
O4—Y1—O7	111.09 (8)	H5A—C5—H5B	109.5
O6—Y1—O7	66.06 (8)	N1—C5—H5C	109.5
O1—Y1—O7	136.38 (8)	H5A—C5—H5C	109.5
O2^i^—Y1—O7	70.27 (8)	H5B—C5—H5C	109.5
O1W—Y1—O7	141.16 (8)	N1—C6—H6A	109.5
O8—Y1—O7	124.12 (8)	N1—C6—H6B	109.5
C1—O1—Y1	117.95 (18)	H6A—C6—H6B	109.5
C1—O2—Y1^ii^	117.94 (19)	N1—C6—H6C	109.5
C2—O3—Y1^ii^	118.85 (19)	H6A—C6—H6C	109.5
C2—O4—Y1	119.03 (19)	H6B—C6—H6C	109.5
			
O3^i^—Y1—O1—C1	−146.8 (2)	O3^i^—Y1—O8—C4	87.9 (2)
O9—Y1—O1—C1	141.7 (2)	O9—Y1—O8—C4	−5.14 (19)
O4—Y1—O1—C1	−9.2 (2)	O4—Y1—O8—C4	−139.29 (19)
O6—Y1—O1—C1	66.0 (2)	O6—Y1—O8—C4	−46.0 (2)
O2^i^—Y1—O1—C1	−26.0 (3)	O1—Y1—O8—C4	−94.3 (2)
O1W—Y1—O1—C1	−82.0 (2)	O2^i^—Y1—O8—C4	129.6 (2)
O8—Y1—O1—C1	−150.3 (3)	O1W—Y1—O8—C4	177.4 (2)
O7—Y1—O1—C1	88.5 (3)	O7—Y1—O8—C4	40.2 (2)
O3^i^—Y1—O4—C2	156.4 (2)	O3^i^—Y1—O9—C4^iv^	−65.9 (2)
O9—Y1—O4—C2	−35.0 (3)	O4—Y1—O9—C4^iv^	122.1 (2)
O6—Y1—O4—C2	−67.7 (2)	O6—Y1—O9—C4^iv^	154.1 (2)
O1—Y1—O4—C2	11.9 (2)	O1—Y1—O9—C4^iv^	79.1 (2)
O2^i^—Y1—O4—C2	179.9 (3)	O2^i^—Y1—O9—C4^iv^	−113.6 (2)
O1W—Y1—O4—C2	100.4 (2)	O1W—Y1—O9—C4^iv^	8.5 (3)
O8—Y1—O4—C2	58.5 (3)	O8—Y1—O9—C4^iv^	5.23 (19)
O7—Y1—O4—C2	−121.0 (2)	O7—Y1—O9—C4^iv^	−136.5 (2)
O3^i^—Y1—O6—C3	2.2 (3)	Y1—O1—C1—O2	−173.7 (3)
O9—Y1—O6—C3	68.0 (2)	Y1—O1—C1—C2	6.4 (4)
O4—Y1—O6—C3	−133.8 (3)	Y1^ii^—O2—C1—O1	−172.3 (3)
O1—Y1—O6—C3	154.5 (3)	Y1^ii^—O2—C1—C2	7.6 (4)
O2^i^—Y1—O6—C3	−69.7 (2)	Y1—O4—C2—O3	167.4 (2)
O1W—Y1—O6—C3	−151.3 (2)	Y1—O4—C2—C1	−13.2 (4)
O8—Y1—O6—C3	106.6 (2)	Y1^ii^—O3—C2—O4	165.7 (3)
O7—Y1—O6—C3	−8.7 (2)	Y1^ii^—O3—C2—C1	−13.8 (4)
O3^i^—Y1—O7—C3^iii^	−163.4 (3)	O1—C1—C2—O4	4.4 (4)
O9—Y1—O7—C3^iii^	−71.8 (2)	O2—C1—C2—O4	−175.5 (3)
O4—Y1—O7—C3^iii^	64.3 (3)	O1—C1—C2—O3	−176.1 (3)
O6—Y1—O7—C3^iii^	8.5 (2)	O2—C1—C2—O3	4.0 (4)
O1—Y1—O7—C3^iii^	−15.1 (3)	Y1—O6—C3—O7^iii^	−171.9 (3)
O2^i^—Y1—O7—C3^iii^	123.9 (3)	Y1—O6—C3—C3^iii^	8.3 (5)
O1W—Y1—O7—C3^iii^	149.8 (2)	Y1—O8—C4—O9^iv^	−175.3 (2)
O8—Y1—O7—C3^iii^	−115.3 (2)	Y1—O8—C4—C4^iv^	4.7 (4)

Symmetry codes: (i) −*x*+1, *y*+1/2, −*z*+1/2; (ii) −*x*+1, *y*−1/2, −*z*+1/2; (iii) −*x*+1, −*y*+2, −*z*+1; (iv) −*x*+2, −*y*+2, −*z*+1.

### Hydrogen-bond geometry (Å, °)

**Table:**

*D*—H···*A*	*D*—H	H···*A*	*D*···*A*	*D*—H···*A*
O1W—H1WA···O2W^iv^	0.83 (2)	1.93 (2)	2.742 (4)	167 (4)
O1W—H1WB···O2W^v^	0.72 (2)	2.20 (2)	2.861 (4)	152 (4)
O2W—H2WA···O6^vi^	0.81 (2)	2.38 (2)	3.143 (4)	158 (5)
O2W—H2WA···O7^vii^	0.81 (2)	2.45 (5)	2.944 (4)	121 (5)
O2W—H2WB···O3W^viii^	0.79 (2)	2.38 (3)	2.963 (7)	131 (4)
O2W—H2WB···O4W	0.79 (2)	2.44 (3)	3.194 (6)	159 (5)
O3W—H3WA···O2	0.88 (2)	2.36 (7)	2.830 (5)	114 (6)
O3W—H3WB···O4W^vii^	0.86 (2)	1.90 (3)	2.735 (6)	161 (6)
O4W—H4WA···O1^ix^	0.82 (2)	2.13 (2)	2.943 (4)	172 (5)
O4W—H4WB···O3^x^	0.83 (2)	2.08 (3)	2.857 (4)	155 (6)
N1—H1A···O8^ix^	0.90	2.00	2.869 (4)	163
N1—H1A···O1W^ix^	0.90	2.54	3.107 (4)	122
N1—H1B···O3W	0.90	1.90	2.784 (6)	166

Symmetry codes: (iv) −*x*+2, −*y*+2, −*z*+1; (v) *x*−1, *y*, *z*−1; (vi) *x*+1, −*y*+3/2, *z*+1/2; (vii) −*x*+2, *y*−1/2, −*z*+3/2; (viii) −*x*+2, *y*+1/2, −*z*+3/2; (ix) *x*, −*y*+3/2, *z*+1/2; (x) *x*+1, *y*, *z*+1.
